# Ensemble learning prediction model for intraoperative hemodynamic instability in patients with pheochromocytoma

**DOI:** 10.3389/fendo.2025.1670909

**Published:** 2025-12-12

**Authors:** Yingshu Liu, Chao Liu, Shen Li, Liang Zhao, Rui Lin, Kang Chen, Li Zang, Weijun Gu, Yiming Mu, Zhaohui Lyu, Zhengnan Gao, Jingtao Dou

**Affiliations:** 1 Department of Endocrinology, The First Medical Center of People's Liberation Army General Hospital, Beijing, China; 2 Department of Endocrinology, Central Hospital of Dalian University of Technology, Dalian, Liaoning, China; 3 Department of Neurology, Johns Hopkins Hospital, Baltimore, MD, United States; 4 Yidu Cloud Technology Inc., Beijing, China; 5 School of Software Technology, Dalian University of Technology, Dalian, Liaoning, China

**Keywords:** ensemble learning, prediction model, hemodynamic instability, intraoperative risk prediction, pheochromocytoma, web-based calculator

## Abstract

**Background:**

Accurately predicting intraoperative hemodynamic instability (HI) in patients with pheochromocytoma is essential for improving prognosis; however, clinically applicable large-sample, high-precision predictive models remain limited. This study develops and validates an ensemble learning (EL) model to predict HI risk.

**Methods:**

This cohort study included a derivation cohort (n = 353) and an external validation cohort (n = 51), from January 2011 to February 2023. General clinical and intraoperative hemodynamic data were collected. Ensemble feature selection was used to identify key predictors. 5-fold cross-validation was repeated 1000 times to develop the EL model. Shapley Additive Explanations was used to analyze feature contributions, and the model was implemented as a web calculator. The primary outcome was the occurrence of intraoperative HI, evaluated by area under the curve (AUC), sensitivity, specificity, and calibration.

**Results:**

Of 45 variables, tumor size, preoperative systolic blood pressure, age, fasting plasma glucose, and body mass index were top predictors. The developed EL model achieved AUC, sensitivity, and specificity values of 0.886, 0.776, and 0.836 and 0.744, 0.733, and 0.667 in Training set and external validations, respectively. Higher SBP (≥ 125 mmHg), larger tumor size (≥ 60 mm), older age (≥ 55 years), higher FPG (≥ 6 mmol/L), and BMI <22 or >30 kg/m² increased HI risk. The model demonstrated strong calibration and is accessible at http://60.205.91.235/.

**Conclusions:**

This study identified five key predictors of intraoperative HI in patients with pheochromocytoma. The developed EL model provides an accurate, clinically applicable HI risk estimation tool, potentially improving clinical management.

## 1 Introduction

Pheochromocytoma is a rare neuroendocrine tumor characterized by excessive catecholamine production, storage, and secretion (1). Surgery currently remains the only effective treatment; however, several factors, such as general anesthesia during surgery, ventilator-mediated abdominal pressure fluctuations, and direct treatment of the tumor, can trigger catecholamine release, leading to intraoperative hypertensive crises. Severe cases may result in stroke, myocardial infarction, heart failure, multiple organ failure, or mortality (2–4). Advancements in preoperative preparation, anesthesia, and surgical techniques for pheochromocytoma have significantly reduced perioperative mortality rates from 20%–45% to 0%–2.9% (5–12). Despite these improvements, hemodynamic instability (HI) still affects 39.1–100% of patients (13–18).

Accurately predicting HI before surgery and implementing timely interventions can significantly reduce its incidence, mitigate complications, and improve surgical outcomes. While current clinical research primarily investigates risk factors for HI, few studies have focused on predictive models for perioperative HI risk or complications in pheochromocytoma cases (12, 14, 19). Existing studies are notably limited by small sample sizes, challenges in acquiring model variables, and the absence of external validation or advanced analytical approaches such as machine learning (ML). This underscores the need for data-driven research utilizing larger sample sizes and more sophisticated analytical techniques.

Compared to conventional statistical models, ML offers superior classification and regression capabilities, effectively handling complex data relationships while simulating the comprehensive diagnostic reasoning of clinicians. Ensemble learning (EL) further enhances predictive performance by integrating multiple ML models. It constructs an optimal predictive framework by leveraging the strengths of diverse algorithms, thereby improving stability and prediction accuracy.

This study employed an EL prediction model to analyze the risk factors for intraoperative HI in patients with pheochromocytoma and validated model performance on an external dataset. The findings provide a foundation for optimizing preoperative preparation, reducing intraoperative HI incidence, and ultimately improving patient outcomes.

## 2 Methods

### 2.1 Patients

This retrospective study included data from 439 patients with pheochromocytoma who underwent unilateral laparoscopic adrenalectomy at the First Medical Centre of the Chinese PLA General Hospital (Beijing, China) between January 2011 and November 2021. Additionally, for external validation, data from 61 patients with pheochromocytoma who underwent the same procedure at the affiliated Central Hospital of the Dalian University of Technology (Dalian, China) between January 2011 and February 2023 were included.

Ethical approval was obtained from the clinical research ethics committees of both hospitals (S2022-085–01 and YN2021-023-01). Due to the retrospective nature of the study, the requirement for informed consent was waived. This study adhered to the principles outlined in the Helsinki Declaration. As inclusion criteria, patients should have been diagnosed with unilateral pheochromocytoma of the adrenal gland, underwent laparoscopic adrenalectomy performed for an adrenal tumor, and received postoperative histopathological confirmation of pheochromocytoma. Exclusion criteria included patients with paraganglioma, malignant pheochromocytoma, a family history of pheochromocytoma-related diseases, or concurrent tumors. Preoperative management included the administration of alpha-blockers and, for some patients, a combination of beta-blockers or even calcium channel blockers for blood pressure control, along with fluid infusion for volume expansion.

Ultimately, 353 patients from the First Medical Centre of the Chinese PLA General Hospital were included in the modelling cohort, while 51 patients from the affiliated Central Hospital of the Dalian University of Technology comprised the external validation set.

### 2.2 Characteristics and outcomes

#### 2.2.1 Case data collection

Clinical data, including symptoms, preoperative medication use, medical history, surgical history, and family history were retrospectively retrieved from electronic medical records of the hospital upon patient admission. Catecholamine and metabolite levels were measured using high-performance liquid chromatography for 24 h urinary catecholamines, including norepinephrine, epinephrine, and dopamine. High-performance liquid chromatography-tandem mass spectrometry was employed to measure catecholamine metabolites, including normetanephrine (NMN), metanephrine (MN), and 3-methoxytyramine. Details regarding testing times and equipment are in **Supplementary Table 1**. Imaging results from computed tomography (CT), magnetic resonance imaging (MRI), and positron emission tomography-CT scans were accessed via the hospital electronic records, documenting tumor location, morphology, and maximum diameter. The tumor size was defined based on the largest diameter identified in the imaging results.

#### 2.2.2 Intraoperative hemodynamic data collection

Perioperative nursing records, temperature charts, and anesthesia records were reviewed to document intraoperative and perioperative complications, surgical techniques, surgery duration, intraoperative hemodynamics (blood pressure and heart rate [HR]), and fluid input and output (crystalloids, colloids, blood products, drainage, and urine output) during and after surgery.

Preoperative blood pressure and HR were measured on the morning of surgery. Intraoperative monitoring included electrocardiography and pulse oximetry, with blood pressure and HR automatically and continuously recorded every 5 minutes via a radial artery catheter. Based on prior studies (11, 20–22), intraoperative hemodynamic characteristics were evaluated using the cumulative frequency and duration of the following events: systolic blood pressure (SBP) ≥ 200 mmHg, SBP ≥ 180 mmHg, SBP ≥ 160 mmHg, SBP < 90 mmHg, diastolic blood pressure (DBP) < 50 mmHg, mean arterial pressure < 60 mmHg, tachycardia (HR ≥ 110 bpm), and bradycardia (HR ≤ 50 bpm).

### 2.3 Model development and evaluation

#### 2.3.1 Model overview

In medical research, EL methodologies provide a refined approach to decision-making processes. Within this framework, the base-learner outcomes resemble the collective assessments of diverse medical practitioners regarding the health status of the patient, while the meta-learner integrates these varied assessments into a cohesive final evaluation. The EL predictive model in this study comprises three core components: feature engineering, base learning, and meta-learning.

#### 2.3.2 Data processing

Data on 73 characteristics from each patient in the derivation cohort were collected. Categorical variables such as diabetes mellitus, hypertension, and the primary outcome variable (HI) were encoded as 0 (not occurring, negative) and 1 (occurring, positive). Owing to significant changes in catecholamine detection methods over the past decade, variables related to catecholamines could not be treated as continuous variables, which is consistent with previous pheochromocytoma-related studies (14). Instead, these variables were encoded as 0 (not elevated) and 1 (elevated) and subsequently combined into composite variables.

The missing proportions of features in the original dataset were analyzed. Features with missing proportions >10% were excluded, while those with <10% were imputed using multiple imputation methods.

Based on the complete dataset, the derivation cohort was randomly divided into a training set and an internal validation set in an 8:2 ratio. This process was repeated 1000 times for robust model evaluation.

### 2.4 Feature selection

To enhance feature screening applicability and identify relevant input features, we carried out ensemble feature selection (EFS) using random forest (RF), adaptive boosting (AdaBoost), gradient boosting decision tree (GBDT), and extremely randomized trees (Extra-Trees) algorithms (23). To minimize the impact of dataset partitioning on feature screening, a 5-fold cross-validation method was applied. The top five features were selected as inputs for the EL prediction model.

### 2.5 Selection of base and meta-learners

The optimal parameters of the RF, AdaBoost, GBDT, Extra-Trees, and Lasso models were determined via grid search. For learner selection, the top five features were used as inputs for the RF, AdaBoost, GBDT, Extra-Trees, and Lasso models, and all the features were used for the Lasso model. Model performance was evaluated using 5-fold cross-validation on the training set. Standard performance metrics, including the area under the curve (AUC), F1 score, sensitivity, specificity, accuracy, precision, positive predictive value, and negative predictive value, were employed.

### 2.6 Model evaluation

The top three models based on performance were selected as candidate-based learners, with the best-performing model serving as the meta-learner to construct an integrated prediction framework. Base learner outputs served as inputs to the meta-learner. The EL prediction models were evaluated on the training and internal validation sets, with the optimal one selected as the final EL prediction model. An external validation set was used to further validate the model. The Shapley Additive Explanations (SHAP) dependence method was used to analyze the relationships between features and their contributions to the HI risk (24). Additionally, a calibration curve assessed the relationship between predicted and actual values. Finally, the EL prediction model was presented as a web calculator.

### 2.7 Model construction and statistical analyzes

Continuous variables with normal distributions were compared using unpaired two-tailed t-tests, while non-normally distributed variables were analyzed using Wilcoxon rank-sum tests. Categorical variables were compared using Chi-squared tests. Model construction and evaluation metrics calculations were performed using Python 3.8 software and libraries, including “sklearn.ensemble,” “sklearn.model_selection,” and “sklearn.metrics.” One thousand random dataset partitions were generated using the train_test_split function in the sklearn.model_selection package, with the current time as the random seed. The statistical significance level was set to *P* < 0.05. Statistical analyzes were performed using R version 4.0.1 software (R Foundation for Statistical Computing, Vienna, Austria) and the “randomForest,” “ggplot2,” and “rms,” packages.

## 3 Results

### 3.1 Dataset

The derivation cohort included 353 patients (183, 51.8% positive). The dataset was randomly divided into training and internal validation sets of 282 and 71 patients, respectively. The external validation set included 51 patients (30, 58.8% positive). **Table 1** presents the clinical and biological characteristics of pheochromocytoma patients with and without HI.

**Table 1:** Clinical and biological characteristics of patients with pheochromocytoma with and without hemodynamic instability (HI).

	With HI	Without HI	*P* Value
Total patients, No.	183	170	
No. of Males/Females	83/100	89/81	0.227
Age at diagnosis, mean (SD), years	50(13.8)	44.9(12.7)	<0.001
BMI, mean (SD), kg/m^2^	24.1(3.6)	24.3(3.5)	0.366
FPG, median (IQR), mmol/L	5.5(4.8,6.6)	5.2(4.7,5.8)	0.005
Hypertension, *n* (%)	135(73.8)	101(59.4)	0.006
Asymptomatic, *n* (%)	28(15.3)	49(28.8)	0.003
Localization of tumor, left/right	92/91	73/97	0.203
Tumor size, median (IQR), mm	51(36,72.5)	41.0(32.0,52.8)	<0.001
Mean urinary, *n*	113	97	
Norepinephrine, median (IQR), μg/24 h	232.2(128.3,439.4)	153.4(60.7,303.5)	0.001
Epinephrine, median (IQR), μg/24 h	55.6(21.0,161.0) ^a^	32(13.7,97.4)	0.055
Dopamine, median (IQR), μg/24 h	379.5(281,554.4)	364(247.0,532.7)	0.453
Mean plasma, *n*	69	54	
Plasma normetanephrine, median (IQR), nmol/L	4.9(1.9,10.1)	2.7(0.9,5.6))	0.02
Plasma metanephrine, median (IQR), nmol/L	0.7(0.1,1.8)	0.1(0.1,0.5)	<0.001
Plasma 3-methoxytyramine, median (IQR), nmol/L	0.8(0.6,0.8) ^b^	0.8(0.8,0.8) ^c^	0.575
ASA degrade, *n* (%)			0.236
1	6(43.3)	12(7.1)	
2	143(78.1)	130(76.5)	
3	34(18.6)	28(16.5)	
Preparation, *n* (%)	179(97.8)	158(92.9)	0.052
Preoperative SBP, median (IQR), mmHg	126(120,136)	120(112,128)	<0.001
Preoperative DBP, median (IQR), mmHg	75(70,80)	72(68,78)	0.001
Operation time, median (IQR), minutes	120(90,150)	114.5(90,140)	0.15

SD, standard deviation; IQR, interquartile range; BMI, body mass index; FPG, fasting plasma glucose; ASA, American Society of Anesthesiologists; SBP, systolic blood pressure; DBP, diastolic blood pressure; a, data were available for 111 patients; b, data were available for 37 patients; c, data were available for 35 patients.

### 3.2 Missing data imputation

The dataset included 73 features, with missing data percentages ranging from 1.13% to 79.06%. Features with missing data rates exceeding 10% (28 features) were excluded, while those with missing rates below 10% (three features) were imputed using multiple imputation techniques. **Supplementary Figure 1** shows the specific missing feature rates.

After imputation, no significant outliers were observed in the feature set. The collinearity correlation matrix heatmap in **Supplementary Figure 2** shows the Pearson correlation coefficients between each pair of variables, with colors representing correlation strength.

### 3.3 EFS

**Figure 1:**
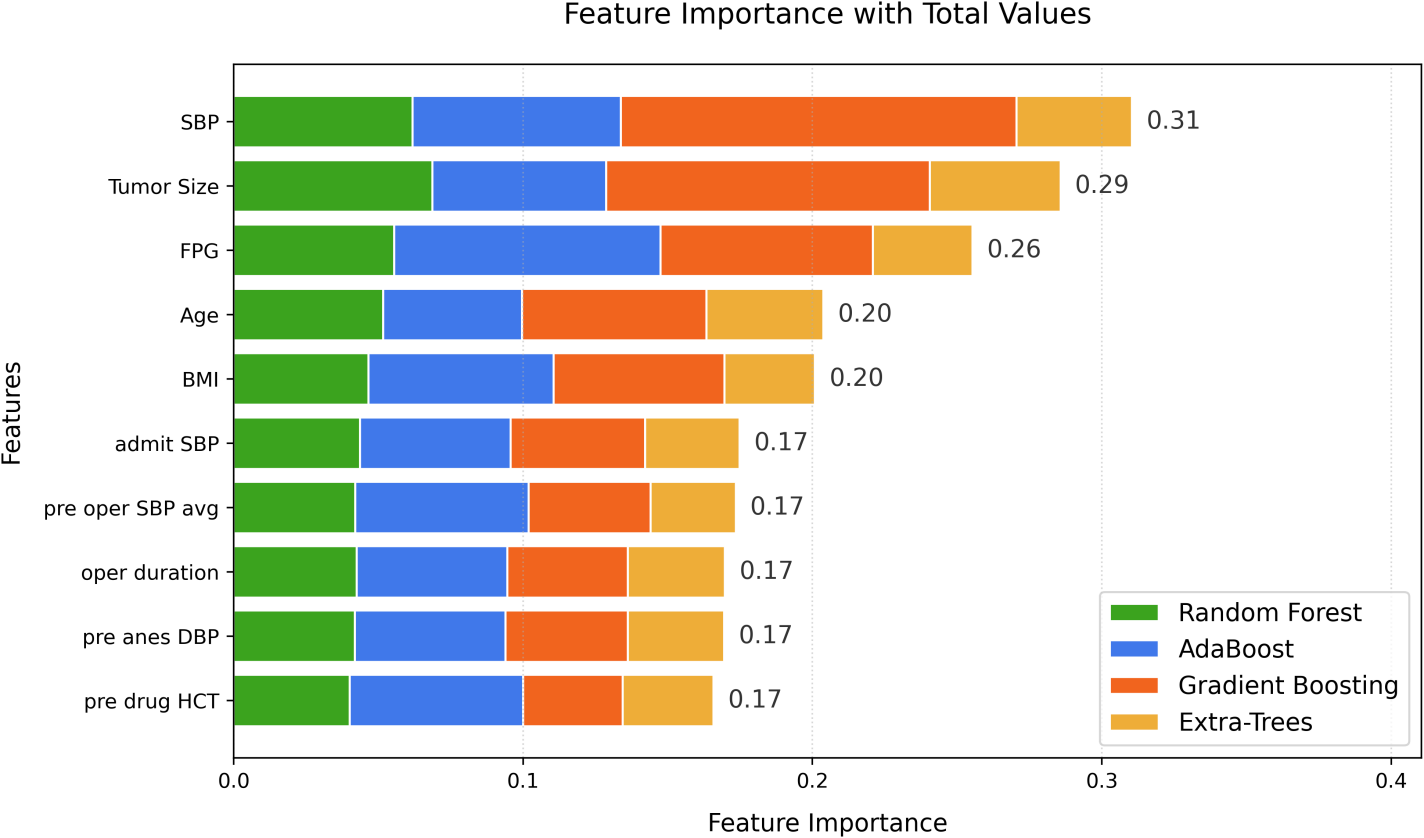
Variables ranking based on ensemble feature selection (EFS) scores. The y-axis shows the top ten variables ranked by importance, while the x-axis shows their cumulative importance values calculated using ensemble feature selection methods such as Random Forest, AdaBoost, Gradient Boosting, and Extremely randomized trees (Extra-Trees).

The feature importance scores were summed and ranked (**Figure 1**), yielding importance scores for 45 features of each model. The top five features, preoperative SBP, tumor size, baseline fasting plasma glucose (FPG), age, and body mass index (BMI), were selected for training the learners.

### 3.4 Base and meta-learners

Among the evaluated models, Extra-Trees yielded the best overall performance, followed by the RF model, which employs a similar generation process. The five-feature Lasso method outperformed the all-feature Lasso approach and ranked third. These top three models served as candidate base learners and were combined into meta-learners with various combinations of base learners. The following EL prediction models were implemented: ET-RF, which employs Extra-Trees and RF as base learners, with Extra-Trees as the meta-learner; ET-LA, which uses Extra-Trees and Lasso as base learners, with Extra-Trees as the meta-learner; and ET-LA-RF, which integrates Extra-Trees, Lasso, and RF as base learners, with Extra-Trees as the meta-learner. All model performances were compared using the metrics listed in **Supplementary Table 2**.

### 3.5 Selection of prediction models

**Figure 2:**
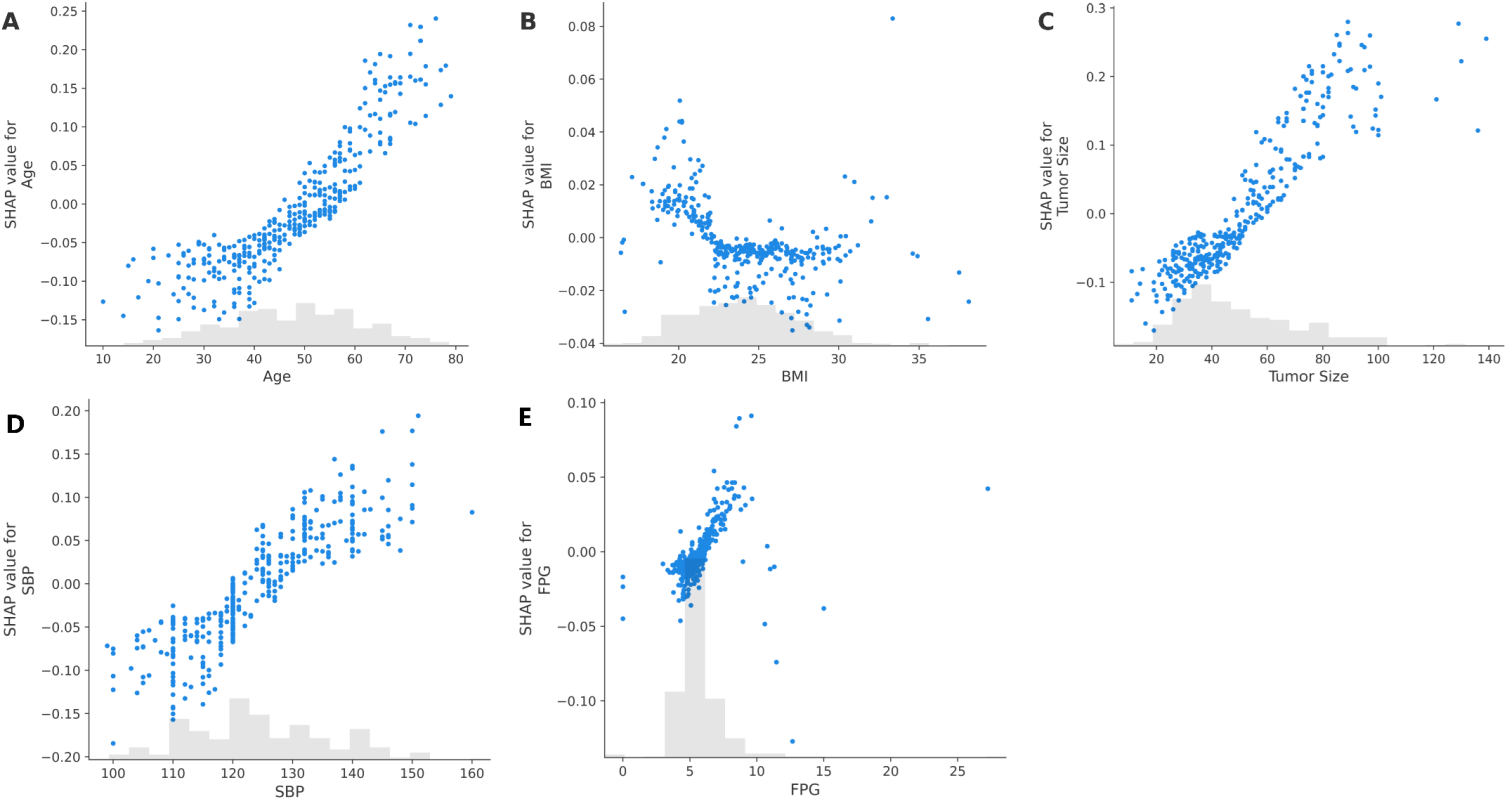
Shapley additive explanations (SHAP) dependence plots for clinical variables predicting hemodynamic instability in patients with pheochromocytoma. The plots show SHAP values for **(A)** Age, **(B)** Body Mass Index (BMI), **(C)** Tumor Size (maximum CT/MRI measurement), **(D)** Systolic Blood Pressure (SBP) before anesthesia, and **(E)** Fasting Plasma Glucose (FPG) before drug administration. Positive SHAP values indicate that higher variable values are associated with an increased risk of hemodynamic instability (HI), while negative values suggest a protective or reduced risk.

Three integrated learning prediction models were compared using 1000 randomly divided training and internal validation sets (**Table 2**). The ET-RF model had the highest AUC (0.886) and the best sensitivity, positive predictive value, and all evaluation indices. On the external validation set, ET-RF outperformed the other two EL prediction models in seven out of eight evaluation metrics (**Table 3**). **Figure 2** shows the SHAP dependence plot for the ET-RF model, where each dot represents a patient. The plot depicts how the attributed importance of each variable changes with its value. SHAP values exceeding zero represent increasing HI risk. Key findings from the SHAP analysis reveal that Higher SBP (≥ 125 mmHg), larger tumor size (≥ 60 mm), older age (≥ 55 years), higher FPG (≥ 6 mmol/L), and BMI <22 or >30 kg/m² increased HI risk.

**Table 2:** Performance of ensemble learning prediction models on training set and internal validation set.

	ET-RF	ET-LA	ET-LA-RF
Training set			
AUC	0.886	0.687	0.731
F1	0.803	0.693	0.618
Sensitivity	0.776	0.746	0.613
Specificity	0.836	0.632	0.700
Accuracy	0.805	0.692	0.656
Precision	0.838	0.672	0.642
PPV	0.838	0.672	0.642
NPV	0.783	0.738	0.652
Internal validation set			
AUC	0.714	0.626	0.686
F1	0.688	0.606	0.611
Sensitivity	0.686	0.642	0.599
Specificity	0.690	0.575	0.704
Accuracy	0.685	0.604	0.648
Precision	0.701	0.615	0.672
PPV	0.701	0.615	0.672
NPV	0.675	0.613	0.639

ET-RF, Extra-Trees and RF as base learners, with Extra-Trees as the meta-learner. ET-LA, Extra-Trees and Lasso as base learners, with Extra-Trees as the meta-learner. ET-LA-RF, Extra-Trees, Lasso, and RF as base learners, with Extra-Trees functioning as the meta-learner. AUC, Area Under the Curve; PPV, Positive Predictive Value; NPV, Negative Predictive Value.

**Table 3:** Comparison of ensemble learning prediction models on the external validation set.

	ET-RF	ET-LA	ET-LA-RF
AUC	0.744	0.654	0.652
F1	0.746	0.690	0.698
Sensitivity	0.733	0.667	0.733
Specificity	0.667	0.619	0.476
Accuracy	0.706	0.647	0.627
Precision	0.759	0.714	0.667
PPV	0.759	0.714	0.667
NPV	0.636	0.565	0.556

### 3.6 Web calculator

To facilitate clinical application, we developed an online web calculator tool for physicians. Physicians need only to input the tumor size, preoperative SBP, age, baseline FPG level, and BMI of the patient. The calculator estimates HI risk based on a selected threshold probability: high-risk estimate probability ≥0.5, low-risk estimate probability <0.5. The calculator is accessible at http://60.205.91.235/.

### 3.7 Evaluation of predictive models

**Supplementary Figure 3** shows the calibration curves of the three models for the internal (left) and external validation sets (right). The ET-RF prediction model, which uses Extra-trees and RF as base learners and Extra-trees as a meta-learner, closely matched the actual outcomes.

## 4 Discussion

In this study, we selected a research center with high surgical volume for pheochromocytoma, utilizing a large sample size and rigorous design that included only patients undergoing laparoscopic surgery. Our resulting predictive model demonstrated strong discriminative capability, enabling clinicians to more effectively implement targeted interventions and enhance patient outcomes.

Previous studies indicate that intraoperative HI can lead to perioperative complications and mortality (8, 11, 19). Although our study aligns with prior findings of zero mortality (5, 8, 9), HI resulted in significant adverse events within our cohort, including cardiovascular complications in eight patients, massive blood loss in five, and cerebrovascular complications in three. Additionally, 16 patients required intensive care due to critical HI episodes, further underscoring the clinical significance of this outcome.

While previous studies have investigated predictive models for intraoperative HI in pheochromocytoma patients (12, 14, 19), many have limited generalizability due to small sample sizes, insufficient external validation, difficulties in acquiring comprehensive model features, and neglecting variability in surgical approaches.

Our study addresses these gaps by integrating a robust sample size, comprehensive patient characteristics, and rigorous methodological standards. Our model has several advantages. First, EFS integrates multiple feature selection algorithms, reducing bias from single-algorithm use (25). Second, previous ML models, like Lasso or linear regression, inadequately capture complex feature interactions (19, 26). T We addressed these limitations by employing ensemble learning (EL), involving feature engineering, base learners, and meta-learners to generate predictions. This integrated approach outperforms single models, providing more accurate clinical predictions. Furthermore, given the established influence of anesthesia and surgical team experience on HI risk (27), we conducted external validation with data from multiple institutions, confirming robust predictive performance and broader clinical applicability (25).

This study reveals that age, BMI, tumor size, preoperative SBP, and baseline FPG are independent risk factors for intraoperative HI in patients with pheochromocytoma. Unlike previous models (12, 14, 19), our analysis using SHAP dependence plots revealed significant associations: a higher preoperative SBP (≥125 mmHg) notably increased HI risk, reinforcing prior findings that optimal preoperative blood pressure management is critical for minimizing HI, related complications, and mortality (16, 28, 29). Current guidelines consistently advise maintaining blood pressure within safe ranges to reduce HI risk (3).

Tumor size is a common risk factor for HI (4, 5, 7, 8, 15). Larger pheochromocytomas typically produce and metabolize more catecholamines, resulting in severe hypertension during adrenalectomy (30). Tumor size also emerged as a critical factor; risk transitioned from negative to positive at a threshold of 55–60 mm. Patients with tumors ≥60 mm are known to experience increased intraoperative blood loss compared to those with smaller tumors (31). Larger pheochromocytomas correlate positively with HI risk, partly due to greater intraoperative blood loss from the surgical procedure and rapid reductions in serum catecholamine levels and circulating blood volume (32, 33).

Four prior studies have utilized preoperative data to predict intraoperative HI, identifying age, tumor size, and BMI as significant independent risk factors (12, 14, 19, 29). Additionally, Previous research on risk factors also suggests a correlation between age and HI (16). Our SHAP dependence analysis corroborates these findings, demonstrating that age above 55 years is associated with increased HI risk, consistent with previous reports identifying age >45 years as an independent risk factor for intraoperative hemodynamic instability (14, 34). Furthermore, BMI is both a risk factor for HI and an independent risk factor for severe postoperative and cardiovascular complications (35, 36). The SHAP dependence plot showed that a BMI outside the specific range 22–30 Kg/m^2^, whether higher or lower, was associated with an increased HI risk. Specifically, lower BMI is often associated with a reduced circulating capacity, making patients more prone to HI.

Glucose regulation plays a pivotal role as well. Patients with concurrent diabetes and pheochromocytoma exhibit elevated perioperative HI risk (17, 18). Our findings indicate increased HI risk when baseline FPG elevated. Insulin resistance in these patients, driven by excessive catecholamine secretion, significantly contributes to HI (18, 37, 38). Additionally, elevated blood glucose levels may reflect underlying cardiovascular dysfunction, such as myocardial impairment and arteriosclerosis, thereby exacerbating intraoperative hemodynamic instability (39, 40).

This study has several limitations. First, it was retrospective, and prospective studies are necessary to further evaluate the applicability of the model. Second, because the retrospective period spanned many years, newer detection methods for catecholamines have since emerged, and measurement standards vary across institutions. Variables related to catecholamines and their metabolites were initially converted into categorical variables but were eventually excluded from feature selection due to substantial missing data. Nevertheless, our model still demonstrated excellent predictive performance for hemodynamic instability. Moreover, since catecholamine and their metabolites measurements are complex and not routinely performed in all centers, the ability of our model to achieve high accuracy without relying on these variables enhances its generalizability and practical utility in clinical settings.

In conclusion, this study analyzed a large sample of patients with varied characteristics and identified five key risk factors for intraoperative HI in pheochromocytoma patients. Using an EL-based prediction model, we developed and validated a risk model for intraoperative HI utilizing internal and external datasets. Additionally, we created a user-friendly and accurate web calculator to assess individualized HI risk prior to surgery. This tool enables clinicians to proactively predict HI risk in patients undergoing laparoscopic unilateral pheochromocytoma resection, enabling timely interventions to reduce HI incidence and improve patient outcomes.

## Data Availability

The original contributions presented in the study are included in the article/**Supplementary Material**. Further inquiries can be directed to the corresponding authors.
