# Supplementary material for: Ensemble learning prediction model for intraoperative hemodynamic instability in patients with pheochromocytoma
Source: Front Endocrinol (Lausanne). 2025 Dec 12;16:1670909. doi: 10.3389/fendo.2025.1670909 (PMC12740857; doi:10.3389/fendo.2025.1670909)
Supplement: Supplementary file 1 [file DataSheet1.docx]

**Supplementary Online Content**

Yingshu Liu, Chao Liu, Shen Li, Liang Zhao, Rui Lin, Kang Chen, Li Zang, Weijun Gu, Yiming Mu, Zhaohui Lyu, Zhengnan Gao, Jingtao Dou

Web-Based Prediction of Hemodynamic Instability in Pheochromocytoma: Aiding Personalized Anesthetic Management

**Supplementary Table 1.** Laboratory equipment information

**Supplementary Table 2.** Comparison of 5-fold cross-validation performance of models on 1000 randomly divided training sets

**Supplementary Figure 1.** Missing feature rates

**Supplementary Figure 2.** Heat map of the correlation matrixes

**Supplementary Figure 3.** Calibration curves of the three models for the internal dataset and external dataset.

This supplementary material has been provided by the authors to give readers additional information about their work.

**Supplementary Table 1.** Laboratory equipment information

|  | Reference Range | Unit | Test Period | Instrument | Method |
| --- | --- | --- | --- | --- | --- |
| Norepinephrine | (22.1,75.3) | μg/24 h | 2011.07-2018.08 | Agilent Technologies 1200 series | High-performance liquid chromatography (HPLC) |
| Epinephrine | (1.5,34.5) |  |  |  |  |
| Dopamine | (93.2,470.3) |  |  |  |  |
| Methoxy-Norepinephrine | [0,0.9] | nmol/L | 2016.10-2021.11 | Waters ACQUITY UPLC I-Class IVD/Xevo TQ-S IVD System | High-performance liquid chromatography-tandem mass spectrometry (HPLC-MS/MS) |
| Methoxy-Epinephrine | [0,0.5] |  |  |  |  |
| 3-Methoxytyramine | [0,0.18] |  |  |  |  |

**Supplementary Table 2.** Comparison of 5-fold cross-validation performance of models on 1000 randomly divided training sets

|  | RF | AdaBoost | GBDT | Extra-Trees | Lasso | Lasso（All features） |
| --- | --- | --- | --- | --- | --- | --- |
| Maximum AUC | 0.7481 | 0.7427 | 0.7521 | 0.7570 | 0.6583 | 0.6450 |
| Average AUC | 0.6776 | 0.6482 | 0.6632 | 0.7106 | 0.6012 | 0.5790 |
| Maximum F1 | 0.7553 | 0.7215 | 0.7206 | 0.7445 | 0.7374 | 0.6970 |
| Average F1 | 0.6515 | 0.6366 | 0.6359 | 0.6784 | 0.6174 | 0.6237 |
| Maximum Sensitivity | 0.7713 | 0.6984 | 0.7492 | 0.8258 | 0.9326 | 0.8297 |
| Average Sensitivity | 0.6587 | 0.6338 | 0.6402 | 0.7036 | 0.6668 | 0.6898 |
| Maximum Specificity | 0.7471 | 0.6894 | 0.7215 | 0.7950 | 0.8050 | 0.7286 |
| Average Specificity | 0.6180 | 0.5858 | 0.6020 | 0.5994 | 0.5456 | 0.4680 |
| Maximum Accuracy | 0.7198 | 0.6984 | 0.6984 | 0.7204 | 0.6816 | 0.6347 |
| Average Accuracy | 0.6395 | 0.6114 | 0.6232 | 0.6564 | 0.5930 | 0.5778 |
| Maximum Precision | 0.7410 | 0.7136 | 0.7192 | 0.7352 | 0.7165 | 0.6568 |
| Average Precision | 0.6518 | 0.6251 | 0.6375 | 0.6605 | 0.6198 | 0.5852 |

Abbreviations: AUC, Area Under the Curve; RF, Random Forest; AdaBoost, Adaptive Boosting; GBDT, Gradient Boosting Decision Tree; Extra-Trees, Extremely Randomized Trees.

**Supplementary Figure 1.** Missing feature rates


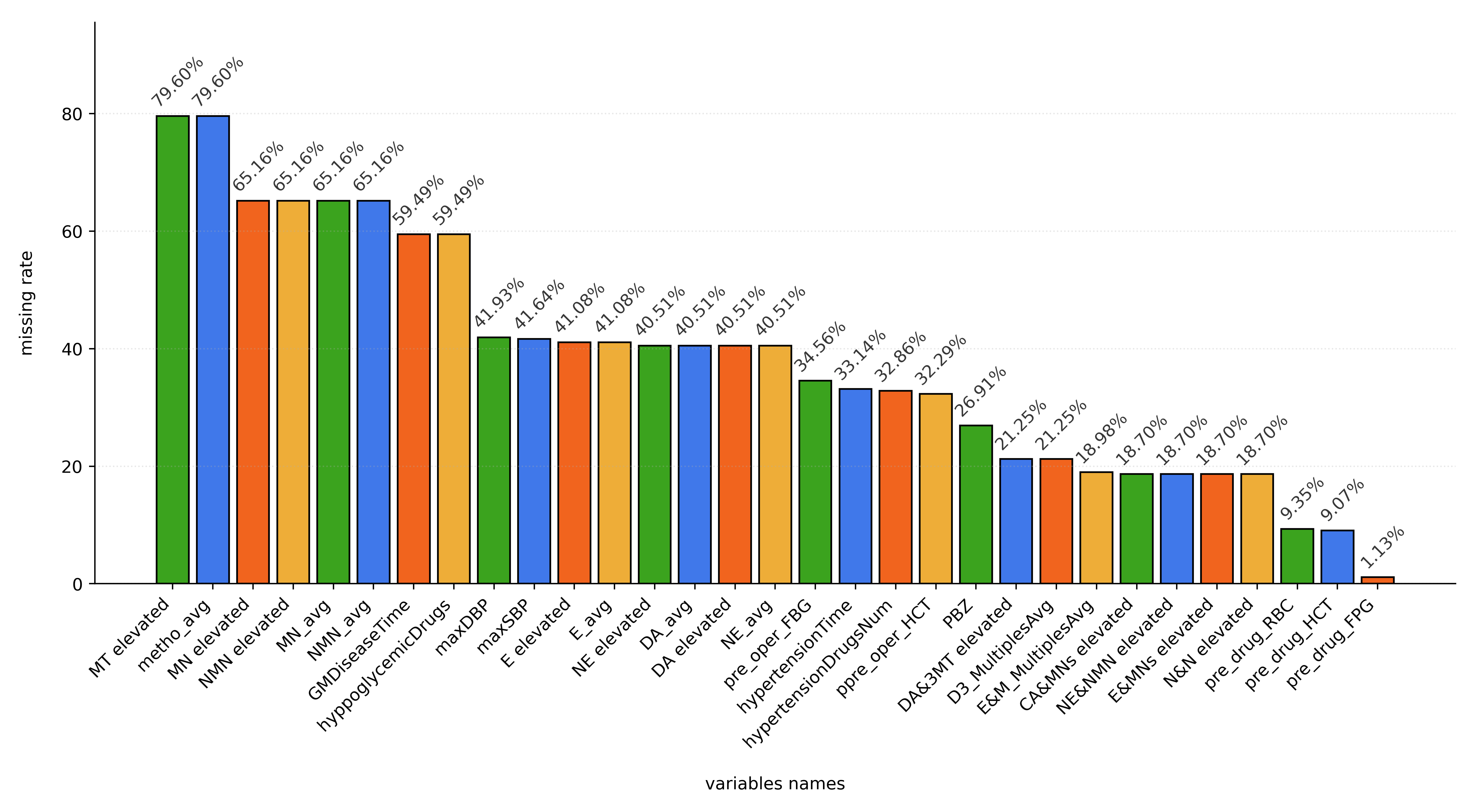


Among the top 31 features with high missing rates, 28 features with missing rates exceeding 10% were excluded from the analysis. For the remaining 3 features with missing rates less than 10%, multiple imputation methods were applied to fill in the missing data.

**Supplementary Figure 2.** Heat map of the correlation matrixes


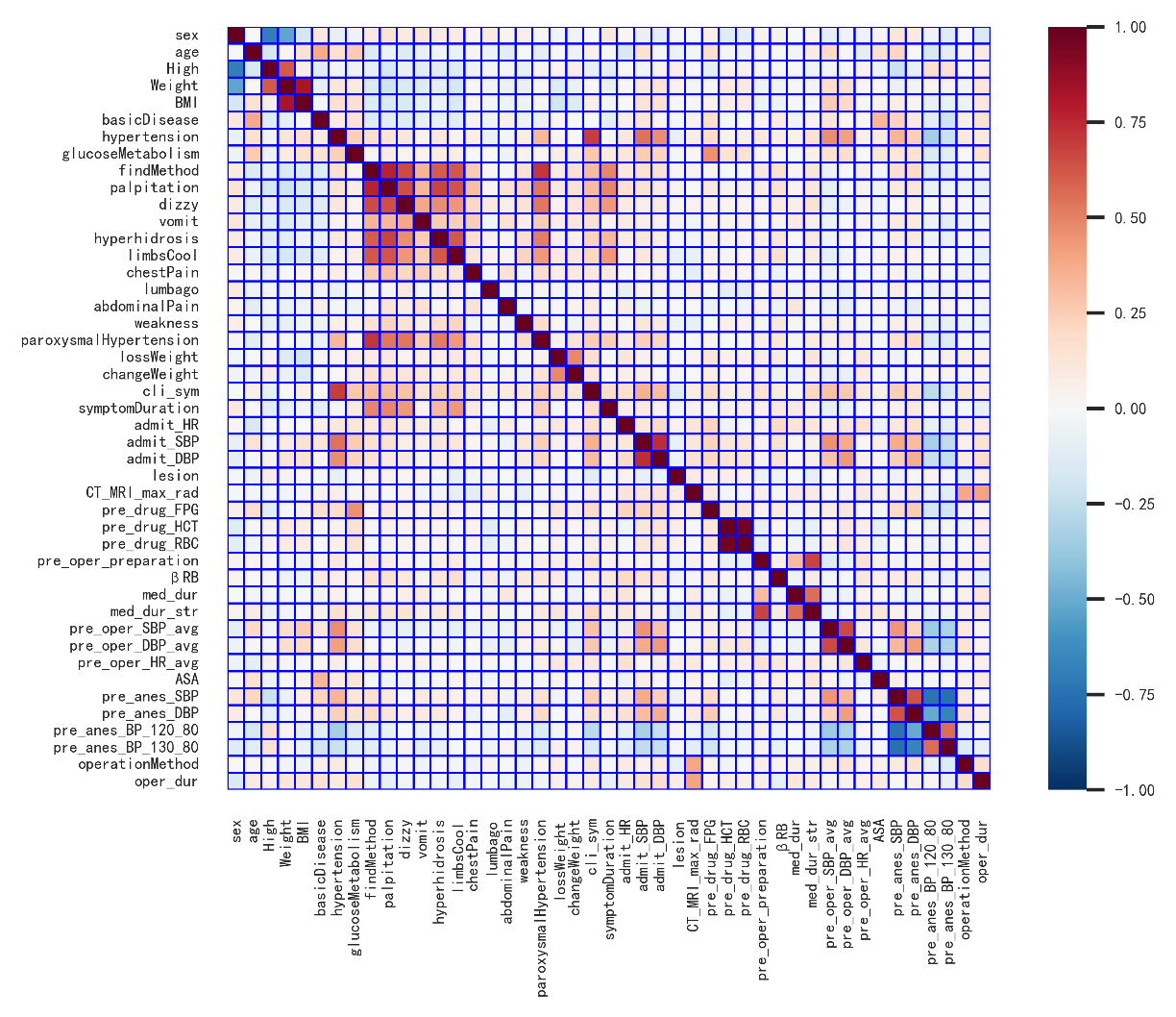


Each cell is filled with a number and is represented by a color. The number indicates the correlation coefficient between the two corresponding variables across all patients. No significant collinearity was found in the data matrix.

**Supplementary Figure 3.** Calibration curves of the three models for the internal dataset and external dataset.


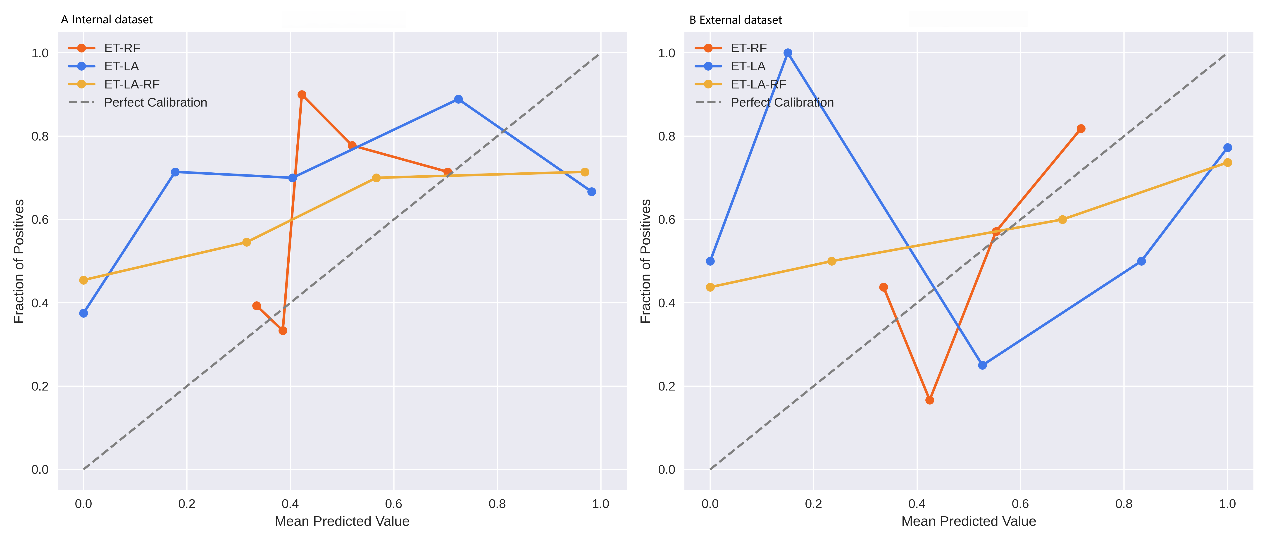


This figure shows the calibration curves of the three models for the internal dataset (left) and external dataset (right). The ER-RF prediction model with Extra-trees and RF as base learners and Extra-trees as a meta-learner closely matched the real situation. Abbreviations: ET-RF employs Extra-Trees and RF as base learners, with Extra-Trees as the meta-learner. ET-LA utilizes Extra-Trees and Lasso as base learners, with Extra-Trees as the meta-learner. ET-LA-RF integrates Extra-Trees, Lasso, and RF as base learners, with Extra-Trees as the meta-learner.
